# Editorial: MR-guided focused ultrasound treatment techniques in cancers: from physics to clinics

**DOI:** 10.3389/fonc.2023.1233833

**Published:** 2023-07-18

**Authors:** Yonggang Lu, Zhongwei Zhang, Shi Liu

**Affiliations:** ^1^ Department of Radiology, Medical College of Wisconsin, Milwaukee, WI, United States; ^2^ Department of Radiology, Washington University School of Medicine, St Louis, MO, United States; ^3^ Department of Medical Physics, Memorial Sloan-Kettering Cancer Center, New York, NY, United States

**Keywords:** MRI, focused ultrasound (MRgFUS), cancers, treatment, diagnosis

Cancer, a formidable adversary affecting countless lives worldwide, demands continuous innovation in treatment methodologies. In recent years, an emerging technology known as MR-guided Focused Ultrasound (MRgFUS) has held tremendous promise in revolutionizing cancer treatment. By seamlessly integrating physics principles with clinical applications, MRgFUS provides a non-invasive and precise approach to targeting tumors. Within this context, on 7/19/2021 we launched the Research Topic ‘*MR-guided Focused Ultrasound Treatment Techniques in Cancers: from Physics to Clinics*’, and invited researchers worldwide to address this remarkable treatment modality and bring up contemporary advancements in this specific area. Despite all hardships, disruption, and uncertainty brought by pandemic, we still received 9 insightful manuscripts from 8 countries and finally 6 manuscripts were accepted after rigorous peer-review. Thanks to all authors and reviewers, with your endeavors and contributions, we are able to delve into this fascinating area and enjoy the exciting advancement of this technique. In this editorial, the contribution of each article is summarized, and the thoughts and perspectives from authors are shared.

The contributions of the six articles can be categorized into three distinct areas of research: (1) Systematic review and insightful perspective of MRgFUS treatment in patients; (2) Technical development and product design in MRgFUS hardware and software system; and (3) Clinical trials with MRgFUS in diagnosing and treating patients.

A first line of research includes contributions of systematic review and insightful perspective of two evolving MRgFUS techniques (transrectal and transurethral) in treating localized prostate cancers (PCa). Both Anttinen et al. and Alabousi and Ghai, based on their previous clinical trial and experience of TULSA (Transurethral Ultrasound Ablation) in treating localized prostate cancers (PCa), addressed challenging issues of current imaging methods in identifying lesion location and evaluating treatment accuracy in patients with PCa, and reviewed recent multicenter phase 2 clinical trials of MRI guided transrectal and transurethral in treating intermediate-risk patients with PCa. It demonstrates that MRI guided transrectal and transurethral treatment is safe and effective, and patients treated with these advanced techniques had better oncologic outcomes.

A second line of research contains an introduction of a novel design of a phased-array transducer with multisegments in focused ultrasound system dedicated to treating transcostal hepatic lesions. Lorton et al. shared their experience in developing the hardware and software of the transducer in their system. Experiments with simulations, tissue mimicking gels, and pigs demonstrated that the transducer had large acoustic windows, strong focusing (within millimeter range), no interference between ultrasound sonication and MR acquisition, and no rib burn or other near-field side effects. It indicates that the system with the new design transducer meets the requirements to perform thermal ablation in deep liver tissues without the need of rib-sparing.

A third line of research is on the sharing of clinical experience with MRgFUS in diagnosing and treating patients. Yeo et al. shared their experience in treating a patient with desmoid tumor at the left dorsal humerus. A four year follow up was performed with an outcome of complete tumor remission and pain relief. In another study, deSouza et al. investigated tissue characteristic of vascular tissues, densely cellular tissues, fat, or bone, and addressed the technical difficulties of delivering HIFU effectively when these tissues are traversed pre-focally or at target. Professional thoughts of treating cancer patients at abdomen/pelvis, brain, bone, breast, and subcutaneous fat were finally provided. Hu et al. explored clinical value of MRI in diagnosis and treatment response evaluation in treating patients with abdominal wall endometriosis (AWE), indicating MRI is an excellent tool for identifying location, size, and concurrent changes of AWE before and after treatment.

Taken together, all the selected studies in our Research Topic bring important and enduring contributions to the understanding of MRgFUS physics, technical development and clinical application. MRgFUS lies the elegant marriage between magnetic resonance imaging (MRI) and focused ultrasound. Utilizing high-intensity ultrasound waves, MRgFUS concentrates energy within the body to precisely heat and destroy tumor cells. The real-time imaging capabilities of MRI offer unparalleled guidance, allowing clinicians to monitor and adjust treatment parameters in response to patient-specific anatomy and tumor characteristics. This amalgamation of physics and clinical expertise paves the way for personalized and precision therapies, minimizing collateral damage and maximizing therapeutic outcomes. Compared to traditional therapies such as surgeries and radiation therapy, MRgFUS offers a host of advantages, such as non-invasive nature, absence of ionizing radiation, faster recovery with minimal discomfort, and alternative treatment options for those patients who may not be eligible for surgery or have previously exhausted standard treatment interventions. By leveraging the power of MRgFUS, clinicians can potentially extend the horizons of cancer care, offering hope to those in need. The potential applications of MRgFUS in oncology are vast and ever-expanding. Breast cancer, uterine fibroids, prostate cancer, and brain tumors are among the many conditions currently being explored for MRgFUS treatment. Additionally, MRgFUS can be utilized for palliative care in patients with painful bone metastases, providing pain relief and improving overall well-being. Promising clinical trials have demonstrated the efficacy and safety of MRgFUS, prompting its integration into mainstream cancer care. As ongoing research continues to unlock its full potential, we can anticipate MRgFUS making significant strides across various cancer types and stages. While MRgFUS has showcased remarkable progress, challenges still persist. Technical limitations, such as treatment time and depth limitations, warrant further refinement. In addition, availability, accessibility, and cost considerations must be addressed to ensure equitable distribution of this transformative technology. Furthermore, long-term follow-up studies are essential to evaluate the durability of treatment response and its impact on overall survival rates. Collaboration among physicists, clinicians, and policymakers is crucial to driving further innovation, optimizing treatment protocols, and expanding the reach of MRgFUS to benefit patients globally.

In conclusion, the convergence of physics and clinical expertise in MRgFUS treatment techniques has ushered in a new era for cancer care. With its non-invasive nature, precise targeting, and expanding clinical applications, MRgFUS represents a paradigm shift in how we approach cancer treatment. As we venture from the realm of physics to the clinics, we must foster collaborative efforts and invest in research and development to fully realize the potential of this groundbreaking technology. By doing so, we can equip clinicians with a powerful tool and offer renewed hope to those battling cancer, steering us closer to a future where the disease is no longer insurmountable.

## Author contributions

YL wrote the first draft of the manuscript. ZZ and SL reviewed and revised the manuscript. All authors listed have made a substantial contribution to the work and approved it for publication.

